# Riluzole as a Neuroprotective Drug for Spinal Cord Injury: From Bench to Bedside

**DOI:** 10.3390/molecules20057775

**Published:** 2015-04-29

**Authors:** Narihito Nagoshi, Hiroaki Nakashima, Michael G. Fehlings

**Affiliations:** 1 Spinal Program, Krembil Neuroscience Center, Toronto Western Hospital, University Health Network, Toronto, ON M5T 2S8, Canada; E-Mails: nagoshi@2002.jukuin.keio.ac.jp (N.N.); hirospine@gmail.com (H.N.); 2 Department of Orthopaedic Surgery, Keio University School of Medicine, Tokyo 160-8582, Japan; 3 Department of Orthopaedic Surgery, Nagoya University Graduate School of Medicine, Nagoya 466-8560, Japan; 4 Department of Surgery, University of Toronto, Toronto, ON M5T 1P5, Canada

**Keywords:** spinal cord injury, riluzole, neuroprotection, clinical trial

## Abstract

Spinal cord injury (SCI) is a devastating event resulting in permanent loss of neurological function. To date, effective therapies for SCI have not been established. With recent progress in neurobiology, however, there is hope that drug administration could improve outcomes after SCI. Riluzole is a benzothiazole anticonvulsant with neuroprotective effects. It has been approved by the U.S. Food and Drug Administration as a safe and well-tolerated treatment for patients with amyotrophic lateral sclerosis. The mechanism of action of riluzole involves the inhibition of pathologic glutamatergic transmission in synapses of neurons via sodium channel blockade. There is convincing evidence that riluzole diminishes neurological tissue destruction and promotes functional recovery in animal SCI models. Based on these results, a phase I/IIa clinical trial with riluzole was conducted for patients with SCI between 2010 and 2011. This trial demonstrated significant improvement in neurological outcomes and showed it to be a safe drug with no serious adverse effects. Currently, an international, multi-center clinical trial (Riluzole in Acute Spinal Cord Injury Study: RISCIS) in phase II/III is in progress with riluzole for patients with SCI (clinicaltrials.gov, registration number NCT01597518). This article reviews the pharmacology and neuroprotective mechanisms of riluzole, and focuses on existing preclinical evidence, and emerging clinical data in the treatment of SCI.

## 1. Introduction

Traumatic spinal cord injury (SCI) is a devastating event, resulting from mechanical disruption of the spinal cord tissue. SCI also occurs in non-traumatic disorders including spondylosis, tumors, and infection. To date, early intervention with surgery, intensive care management to support cardio respiratory systems, and rehabilitation are the cornerstones of treatment for SCI [1,2,3]. However, effective neuroprotective therapies which directly affect the pathobiology of SCI are lacking.

Riluzole acts as a neuroprotective drug to block pathological influx of sodium and inhibit abnormal glutamatergic neurotransmission in the central nervous system (CNS). It can be administrated orally and the U.S. Food and Drug Administration (FDA) has approved this drug as a safe and well-tolerated treatment for patients with amyotrophic lateral sclerosis (ALS). The efficacy of riluzole has been shown, in two randomized controlled trials, to increase tracheostomy-free survival in patients with ALS [4,5]. A 2009 Cochrane review, summarizing the findings of four placebo-controlled, randomized trials, concluded that riluzole is safe and improves median survival by 2–3 months in patients with ALS [6].

In preclinical studies of SCI, riluzole has been shown to improve both neurological and pathological outcomes in injured animals. Based on these results, a phase I clinical trial for SCI was conducted, and results from this validated the efficacy and safety of riluzole administration. Currently, an international, multi-center clinical trial in phase II/III, named Riluzole in Acute Spinal Cord Injury Study (RISCIS), is in progress in patients with SCI (clinicaltrials.gov, registration number NCT01597518). This article reviews the biologic rationale, existing preclinical evidence, and emerging clinical data for the use of riluzole in the treatment of SCI.

## 2. Mechanisms and Pharmacology of Riluzole

### 2.1. Molecular Structure and Mechanisms of Riluzole as Neuroprotective Agent

**Figure 1:**
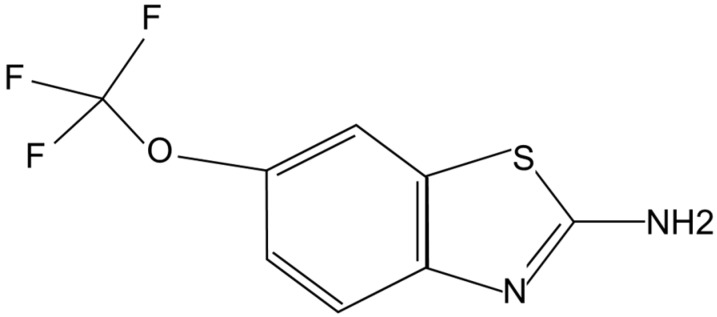
Chemical structure of riluzole.

Riluzole is a capsule-shaped, white, film-coated tablet for oral administration containing 50 mg of riluzole. Chemically it is 2-amino-6-(trifluoromethoxy) benzothiazole. Its molecular formula is C_8_H_5_F_3_N_2_OS and its molecular weight is 234.2. Its structural formula is shown in Figure 1.

**Figure 2:**
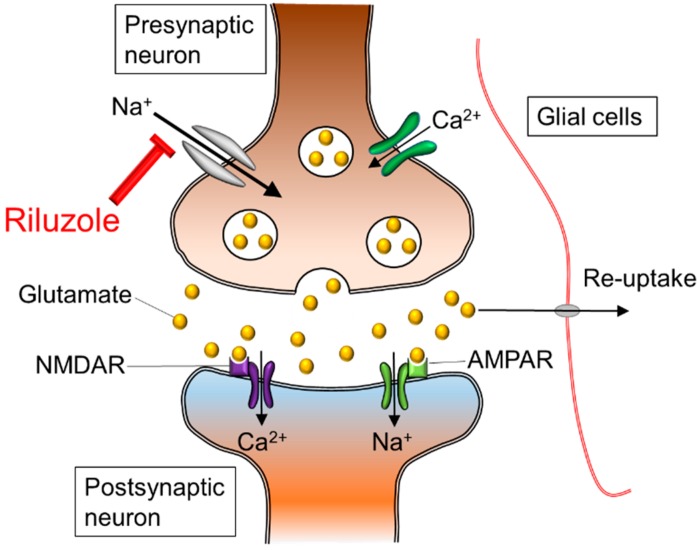
Ionic imbalance and glutamate excitotoxicity. The excessive influx of sodium and calcium triggers extracellular release of glutamate in presynaptic neurons. In the postsynaptic neuron, sodium and calcium influx through NMDAR and AMPAR lead to cellular death and axonal edema. The neuroprotective effects of riluzole appear to result from a blockade of sodium channels, and prevention of exaggerated calcium influx. In addition, riluzole plays a role as an anti-glutamatergic agent via the inhibition of glutamate release in isoxazolepropionic acid receptors: NMDAR; N-methyl-D-aspartic acid receptor, AMPAR; Alpha-amino-3-hydroxy-5-methyl-4-isoxazole propionic acid receptor.

Riluzole is a sodium channel blocker, and its neuroprotective effects for the spinal cord are exerted on neurons and axons to reduce intracellular increases of sodium ion concentration and to reverse operation of axonal sodium calcium exchangers (Figure 2) [7]. Sodium channel blockade may preserve spinal cord white matter by preventing the disruption of the axonal sodium hydrogen antiporter system [8]. In addition, riluzole acts as an anti-glutamatergic agent via the reduction of glutamate release, the prevention of glutamate receptor hypofunction, and the increase of glutamate uptake by activating glutamate transporters and by reducing the release of excitotoxic glutamate [9,10,11]. The multifaceted effects of riluzole on excitotoxicity and neuromodulation make it a promising neuroprotective treatment option for spinal cord disorders and trauma.

### 2.2. Pharmacokinetics of Riluzole

The pharmacokinetics of riluzole have been established in healthy individuals as well as in patients with ALS [12,13,14,15]. Riluzole has been administered intravenously in a single dose of 50 mg and orally in a variety of dose combinations ranging from single doses of 25 mg, 50 mg and 100 mg, incremental doses up to 300 mg and in repeated doses of 25 mg, 50 mg and 100 mg *bis in die* (BID) [13]. Riluzole is rapidly absorbed from the gastrointestinal tract and reaches maximum plasma concentration (C_max_) within approximately 1 h after oral administration [12,13]. Studies show that, when administered at a dose of 50 mg BID, the C_max_ is achieved, on average, at 0.75–0.9 h (SD = 0.5 h) [12,13]. After reaching the C_max_, plasma concentration rapidly declines [12,13]. The steady state through plasma concentrations are achieved at 48–60 h after dosage [13]. At a dosage of 50 mg BID, the terminal elimination half-life was 14.7 h [13].

Riluzole is highly protein bound to serum albumin and lipoproteins (96%), which poses potential concerns for drug-drug interactions with other concomitant medications that compete for protein binding. In patients taking such concomitant medications, a higher concentration of free riluzole in the plasma would be anticipated to exert a greater therapeutic activity.

Riluzole is metabolized in the liver by an enzyme of the cytochrome P450 (CYP) family; specifically by a member of the CYP 1A2 subfamily. The substrates of CYP 1A2 include acetaminophen, caffeine, theophylline, and warfarin [16]. Its inhibitors include tacrine (Cognex), omeprazole (Prilosec), quinolone antibiotics, erythromycin, and oral contraceptives. Co-administration of riluzole with these agents can increase riluzole blood concentrations.

A pivotal, double-blind, randomized, controlled trial of riluzole in patients with ALS investigated the efficacy and safety of riluzole in doses of 25 mg BID, 50 mg BID and 100 mg BID [5]. The dose of 25 mg BID was not significantly better than placebo. Doses of 50 mg BID and 100 mg BID were superior to placebo, with no difference between the two doses. However, adverse events including dizziness, diarrhea, and anorexia were more frequent with the 100 mg BID than with lower doses, and raised aminotransferase activity was dose related [5]. Based on these results, the 50 mg BID was the optimal dose for the treatment of ALS.

## 3. Pathophysiology of SCI and Target for Treatment

**Figure 3:**
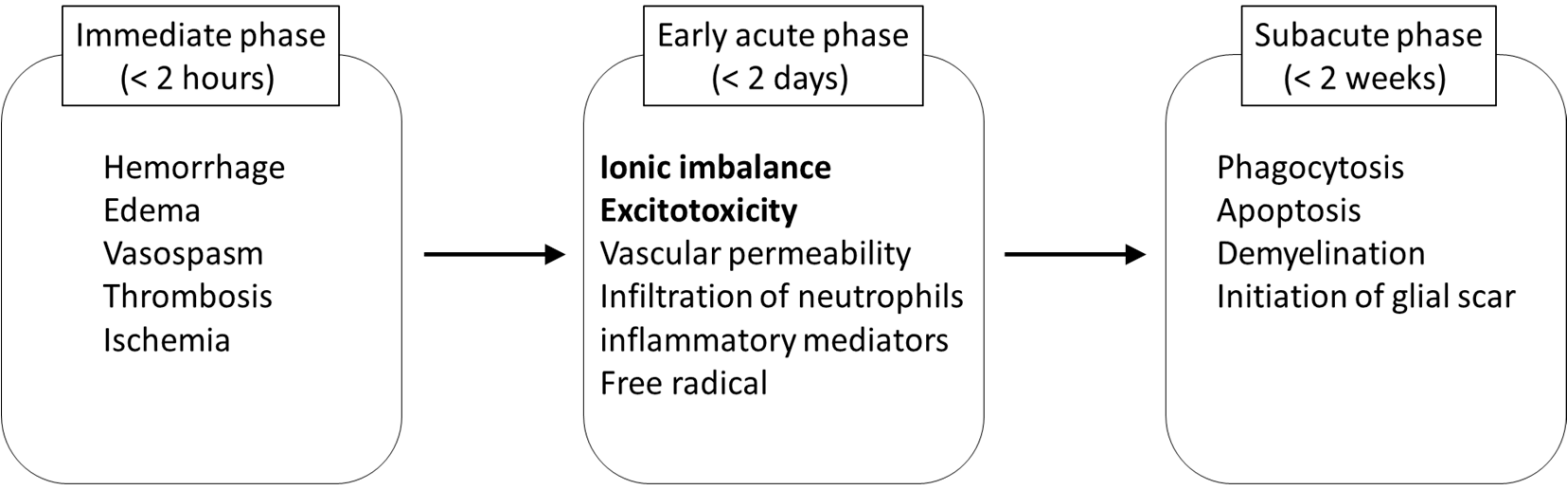
Key pathological events in secondary injury of spinal cord ionic imbalance and excitotoxicity are the targets in riluzole treatment.

The damage to neural tissue from traumatic SCI occurs in two stages, including a primary and a secondary phase. In the primary phase, a complex array of mechanical factors causes the injury to the spinal cord, and neurological dysfunction immediately occurs at and below the level of the injury. The initial mechanical impact and subsequent persisting compression on the spinal cord tissue initiates secondary pathophysiological events which amplify the primary damage (Figure 3). Within a few seconds to minutes after the injury, disruption of microvasculature causes hemorrhage in the grey matter, and edema in the white matter of the spinal cord [17]. These events increase the volume of extracellular fluid, and impair the perfusion of the blood in the injured spinal cord. As a result, thrombosis and vasospasm occur by the release of coagulation factors and vasoactive amines, and ischemic status results [17].

The subsequent secondary damage begins in the early acute phase and is considered to last from 2 h to 2 days after SCI. One of the main events during this stage is ionic dysregulation and excitotoxicity. Neuronal ionic balance is disrupted, and intracellular sodium concentration increases as a result of trauma-induced activation of voltage-sensitive sodium channels (Figure 2) [18,19]. The increases in intracellular sodium concentration also promote concomitant influx of calcium ions through the sodium calcium exchanger, resulting in the development of intracellular acidosis and cytotoxic edema [20,21,22,23]. The excessive influx of sodium and calcium triggers pathologic extracellular release of excitatory neurotransmitter glutamate in presynaptic neurons [24]. High concentrations of glutamic acid can accumulate in the synaptic cleft and trigger an excitotoxic process [25]. The presence of excitotoxic concentrations of glutamic acid in the synaptic cleft results in excessive stimulation of excitatory amino acid receptors on the postsynaptic cell, leading to the entry of sodium and calcium ions through N-methyl-D-aspartic acid (NMDA) and non-NMDA receptors [26,27]. This phenomenon depolarizes the cell and triggers the activation of voltage-dependent sodium and calcium channels, amplifying and spreading the depolarization. This process eventually leads the postsynaptic neural axons to edema and death.

In addition to these pathological conditions, permeability of the blood-brain barrier increases at the early acute stage, and is affected by a number of inflammatory mediators involved in the secondary injury cascade [28]. At the continuing subacute stage in SCI, the delayed secondary injury proceeds by phagocytosis, apoptosis, and demyelination followed by cyst formation [7].

Therefore, one of the major therapeutic strategies for the treatment of SCI is to block the sodium channels to prevent the excessive influx of sodium ions, and to reduce excessive release of glutamic acid. So far, sodium channel blockers, such as lidocaine, phenytoin, and tetrodotoxin, have been shown to attenuate the secondary damage, and promote functional recovery after SCI in animal models [29,30]. Despite the encouraging findings, these compounds have not been progressed to clinical trials.

## 4. Preclinical Studies for SCI with Riluzole

There is convincing evidence from the preclinical realm that riluzole attenuates certain aspects of the secondary injury cascade leading to diminished neurological tissue destruction in animal SCI models. In a series of *in vitro* electrophysiologic experiments from the Fehlings group, the neurophysiologic function of the dorsal column of spinal cords isolated from adult rats were studied after clip compression injury [19]. The authors determined that reduction in extracellular sodium concentrations improved function after injury, whereas increasing axonal membrane permeability to sodium led to reduced function. When the injured axons were cultured with a solution containing sodium channel blocking compounds, an improvement in electrophysiologic function was observed. These results indicate that an increased concentration of intracellular sodium led to a decline in axonal function, and provided an experimental rationale for investigating sodium channel blockade as a means of averting secondary injury after SCI.

Building on the results from the *in vitro* study, the Fehlings group compared the effect of riluzole to phenytoin and CNS5546A, a sodium channel blocker with NMDA antagonistic activity, using a cervical injury model in rats (Table 1) [31]. The extracellular glutamate rises to a toxic level within 15 min after SCI [32] and the drugs were administered at this 15 min time point following SCI [31].

**Table 1:** Preclinical study to examine the efficacy of riluzole for SCI.

References	Animal Species and Injury Level	Injury Model	Timing of Riluzole Administration	Study Summary
Wu *et al.* (2014)	Rats (350–430 g)	Spinal cord ischemia induced by intra-aortic balloon catheter	4 h after injury	Functional recovery was observed in riluzole-treated rats. Riluzole also reduced neuronal loss, infiltration of microglia/macrophages and astrogliosis in the ventral horn and intermediate zone of the gray matter. Riluzole reduced apoptosis of neurons in the dorsal horn of the gray matter.
Wu *et al.* (2013)	Rats (250–300 g) C7-T1	35 g clip	Either 1 or 3 h after injury Twice daily for 7 days	Initiation of riluzole treatment 1 and 3 h post-SCI led to functional and histological recovery. Pharmacokinetic data clarified the prolonged half-life and delayed elimination in the injured rats, suggesting the importance of close monitoring of pharmacokinetics in SCI.
Ates *et al.* (2007)	Rats (200–250 g) T7-10	Weight-drop trauma	Just after injury	In the acute stage, the riluzole and mexiletine treatment groups had better malondialdehyde levels and less spinal cord edema than the phenytoin treatment group. In the chronic stage, riluzole and mexiletine treatment achieved better results for neurobehavioral and histopathological recovery than phenytoin treatment.
Schwartz *et al.* (2001)	Rats (225–280 g) C7-T1	53 g clip	15 min after injury	Neurological recovery was significantly enhanced in animals treated with riluzole compared with the groups treated with other sodium channel blockers. Riluzole significantly reduced tissue loss, and increased red nuclei neurons.
Mu *et al.* (2000)	Rats (225–250 g) T10	NYU impactor	2 and 4 h after injury per each rat Once daily for 7 days	Combination treatment with methylprednisolone was found to significantly improve behavioral outcomes, and promote tissue sparing at the lesion epicenter.
Lips *et al.* (2000)	Rabbits (3.4 ± 0.3 kg)	Spinal cord ischemia induced by infrarenal balloon occlusion of the aorta	15 min before occlusion Twice daily for 3 days	Neurologic outcome was better in the riluzole group compared to controls. Significantly more motor neurons were present in the riluzole group than in controls.
Lang-Lazdunski *et al.* (1999)	Rats (350–400 g)	Spinal cord ischemia induced by aortic cross-clamping	30 min before clamping and at the onset of reperfusion	Riluzole-treated rats had significantly better neurologic function. Histopathology showed that riluzole prevented motor neuron injury, and dramatically attenuated apoptotic neuronal death.
Stutzmann *et al.* (1996)	Rats (260–300 g) T10-12	Balloon catheter in cord canal	30 min after injury Twice daily for 10 days	Animals receiving riluzole recovered motor function, and their somatosensory evoked potential returned towards pre-injury levels. Histological studies revealed significant reduction in the degree of spinal cord infarct after riluzole treatment.

Among these drugs, only riluzole significantly enhanced the functional neurological recovery of coordinated hindlimb function and strength. The functional recovery has been corroborated by histological analysis, indicating significant long-term tissue sparing at and around the site of the injured spinal cord, an increase in the number and size of neurons in the red nucleus, and a reduction of cavity area in riluzole-treated animals. These findings were further explored to investigate optimal timing and duration of administration [33]. The Fehlings group tried administering the riluzole at 1 and 3 h post-injury, and continued for 7 days. The delayed post-injury administration, to 1 and 3 h rather than at 15 min after SCI, also resulted in better sensory-motor function, improved axonal conduction, reduced apoptosis and reduced inflammation without an increase in neuropathic pain. Pharmacokinetic data clarified the prolonged half-life and delayed elimination in the injured rats compared to uninjured ones, reinforcing the importance of close monitoring of pharmacokinetics in SCI patients during clinical trials and the necessary adjustment of riluzole administration accordingly [33]. These results suggest that a reasonable exploratory time window for clinical trial is 12 h post-injury because pathological changes in SCI peak around four times more rapidly in rats than they do in humans [33]. Neuroprotective effects were also observed when riluzole was administered 4 h post-injury in a rat model involving high thoracic aortic balloon occlusion to produce an ischemia/reperfusion injury [34].

Several studies from a number of independent laboratories, in various species of animals, have shown that riluzole is neuroprotective and promotes functional neurological recovery in models of spinal cord ischemic and traumatic injury (Table 1) [35,36,37,38,39]. Riluzole also has an antinociceptive effect and plays a role in spasticity suppression [40,41]. The demonstrated neuroprotective effects of riluzole in various models provide a strong rationale to investigate riluzole in clinical trials.

## 5. Phase I Clinical Trial for SCI Patients

### 5.1. Study Design and Evaluation of Riluzole in Phase I Trial

In the phase I clinical trial (clinicaltrials.gov, registration number NCT00876889) of riluzole for acute SCI, 36 patients (28 cervical and eight thoracic level) were enrolled between April, 2010 and June, 2011 at six clinical centers of the North American Clinical Trials Network (NACTN) [18,42]. The sample size was based on complication rates observed in NACTN registry data [42,43]. Those enrolled were admitted to a NACTN center within 12 h of SCI, and were assessed using the American Spinal Injury Association (ASIA) Impairment Scale (AIS) as grade A, B, or C at admission. For reference, AIS divides SCI into 5 grades—A to E—in which grade A is the most severely damaged spinal cord and grade E indicates normal function without any neurologic deficits [44]. Riluzole (50 mg) was administered every 12 h orally or by nasogastric tube, starting within 12 h of injury for 28 doses. A comparison group was formed of 36 SCI patients who had received standard of care treatment, but did not have riluzole administration. The control patients were matched by AIS grade, gender, and age, so there were no statistically significant differences in demographics or clinical variables for the riluzole and registry patient groups. At time of neurological outcome measurements, the ASIA motor score mean changes from admission to 90 days in cervical injury patients showed significant improvement in the riluzole-treated group compared to the control group (*p* = 0.021). In particular, the greatest gains in mean motor score occurred in grade B patients with cervical injury (*p* = 0.037).

### 5.2. Pharmacokinetics of Riluzole in SCI Patients

In the phase I trial, plasma samples for the pharmacokinetic study were collected one to two hours pre-dose and two hours post-dose for C_max_ and trough concentrations (C_min_), respectively, on days 3 and 14 after the initial dose [45]. Riluzole pharmacokinetics were evaluated in 33 patients on day 3 and in 32 patients on day 14, both C_max_ and C_min_ samples were collected and quantifiable. The plasma concentration and the systemic exposure to riluzole (AUC_0–12_) varied significantly among patients. Mean C_max_, C_min_ and AUC_0–12_ were significantly higher on day 3 than on day 14, resulting from lower clearance and a smaller volume on day 3 [45]. This phenomenon was consistently observed in all patients from all clinical sites.

#### 5.2.1. Adverse Events and Medical Complications

SCI patients have a high incidence of physiological disturbances and medical complications occurring acutely following injury, as documented in a recent publication of data from the North American Clinical Trial Network (NACTN) SCI registry [46]. Using the definitions of moderate and severe complications described in that paper, the incidence of complications occurring within 30 days of injury was carefully monitored during the phase I riluzole study. The frequency of these complications in this study were: infection, including pneumonia (39%); pulmonary, including respiratory failure, lobar collapse, atelectasis and pneumothorax (33%); hematological, including anemia, thrombocytopenia and coagulopathy (22%); cardiac, including arrhythmia and shock (14%); psychiatric, including cognitive decline and depression (14%); gastrointestinal/genitourinary, including severe ileus and hematuria (11%); and skin, including pressure sores (8%). The frequency of these complications was similar to that occurring in patients in the NACTN SCI registry [46]. There were no serious adverse events attributable to riluzole. There was no mortality.

#### 5.2.2. Elevation of Liver Enzymes and Bilirubin

Elevation of liver enzymes has been reported in patients with ALS undergoing treatment with riluzole [5]. Elevation of liver enzymes is also known to occur acutely in patients with SCI, and in animal models of SCI, possibly due to impairment of blood flow to the liver [47,48]. Elevation of alanine transaminase (ALT) and aspartate aminotransferase (AST) are considered to be sensitive indicators of drug-induced damage to liver cells. Elevation of γ-glutamyl transpeptidase (GGT) is a less specific indictor of drug-induced damage to liver cells. Elevation of alkaline phosphatase (ALP) is considered to be primarily an indicator of obstruction of the bile duct.

In this trial, liver enzymes and bilirubin were monitored on days 3, 7, 10 and 14 of administration of riluzole [45]. Elevated levels of liver enzymes and/or bilirubin were found on admission in 9%–37% of patients. Thirteen percent of patients had mild or moderate elevations of ALT, 37% had mild or moderate elevations of AST, 11% had mild elevations of GGT and 9% had mild elevations of bilirubin. Some patients had elevation of a single enzyme, while others had two or three elevated enzymes. The incidence of elevation of enzyme levels increased during the administration of riluzole, with increasing frequency in the second week of administration. In many cases, the elevated concentration had returned to a normal level at the next date of testing. The elevation of one enzyme was not necessarily linked to the elevation of another enzyme. No relationship was found between the C_max_ of riluzole and enzyme levels. In this phase I trial, a placebo-controlled group was not registered, and it still not known how much liver enzymes elevate in patients undergoing treatment with riluzole compared with to a control group of patients. The detailed pharmacodynamics will be clarified in the ongoing IIB/III clinical trial phase.

## 6. Phase IIB/III Randomized Controlled Trial

A phase IIB/III trial started in January 2014 to evaluate the efficacy and safety of riluzole in the management of patients with acute SCI named RISCIS (clinicaltrials.gov, registration number NCT01597518). RISCIS is a randomized, multicenter, placebo-controlled, 2-arm parallel group superiority trial with a sequential adaptive design. This trial is being performed at 17 research hospitals: 14 sites are in the USA, two in Australia and one in Canada. The main inclusion criteria are as follows: (1) SCI with ASIA Impairment Scale Grade “A,” “B” or “C” and neurological level of injury between C4-C8 based upon first ASIA evaluation after arrival to the hospital; (2) aged between 18 and 75 years; (3) able to receive the investigational drug within 12 h of injury. Patients are being enrolled currently, and a total of 11 patients have been enrolled to date in the study (at the time of writing: February, 2015).

### 6.1. Treatment Description

Subjects assigned to the active treatment arm receive riluzole at a dose of 100 mg BID in the first 24 h followed by 50 mg BID for the following 13 days post-injury. The decision to use the 100 mg loading dose was based on the pharmacokinetics and pharmacodynamics results in the Phase I study: large differences in maximal concentration of riluzole between patients were observed and thus, the threshold for efficacy might not be reached in patients with low levels of riluzole. 100 mg BID is an approved FDA dosage and primary riluzole dose was changed to reach optimal therapeutic levels faster. Subjects randomly assigned to the control arm receive a placebo capsule that is identical in shape, size, and color to the riluzole capsule for the same duration and at the same intervals.

### 6.2. The Primary and Secondary Outcomes

As a primary objective of the RISCIS study, neurological motor recovery at 6-month follow-up is compared between adult patients with acute SCI receiving either riluzole or a placebo medication for the same duration after acute SCI. The secondary objectives are to evaluate the impact of this riluzole regimen on sensory recovery, functional outcomes, quality of life outcomes, health utilities, as well as on mortality and adverse event rates.

### 6.3. Pharmacological Sub-Study

The specific aims of this pharmacological sub-study are to determine the individual peak and trough concentrations of riluzole after enteral administration of the study doses described above. This pharmacological sub-study will provide rationale and support for an appropriate approach to the monitoring of riluzole plasma levels and the adjustment of the enteral dose in clinical practice. As a result, individual pharmacokinetic parameters of half-life (t_1/2_), systemic exposure (AUC_0→24_), volume of distribution (Vd) and clearance (CL) will be derived with a one-compartment model, using Bayesian iterative two-stage procedure. Riluzole concentration will also be analyzed in correlation with laboratory measures including AST, ALT, white blood count, and the incidence of adverse events, as well as with neurological outcome scores.

## 7. Conclusions

Basic research into riluzole has a relatively long history and its validation for efficacy in SCI has been demonstrated, leading it to now be investigated in clinical studies. Riluzole is one of several investigational drugs to make the translational leap to clinical trials from preclinical research in SCI [49], and the process it has been through should be referred to for future drugs or molecular compounds intended for use in clinical studies. Although the clinical efficacy of riluzole is still an open question, the results from larger multicenter study will reveal the impact for the treatment of SCI.
